# Design space definition for a stirred single-use bioreactor family from 50 to 2000 L scale

**DOI:** 10.1186/1753-6561-7-S6-P55

**Published:** 2013-12-04

**Authors:** Thomas Dreher, Ute Husemann, Sebastian Ruhl, Gerhard Greller

**Affiliations:** 1 Sartorius Stedim Biotech GmbH, Göttingen, Germany, D-37079

## Background

Single-use bioreactors continue to gain large interest in the biopharmaceutical industry. They are excessively used for mammalian cell cultivations, e.g. production of monoclonal antibodies and vaccines [1]. This is motivated by several advantages of these bioreactors like reduced risk of cross contaminations or shortening lead times [2]. Single-use bioreactors differ in terms of shape, agitation principle and gassing strategy [3]. Hence, a direct process transfer or scale-up between different systems can be a challenge. Reusable bioreactors are still regarded as gold standard due to their well-known and defined geometrical properties. Based on this knowledge a stirred single-use bioreactor family from 50 to 2000 L scale was developed with similar geometrical ratios like commonly used reusable systems. To follow a Quality by Design approach the key process parameters for a modern mammalian cell cultivation were specified. Therefore, the k_L_a-value, mixing time and the power input per volume were evaluated by using process engineering methods for all scales.

## Stirred single-use bioreactor family

The used stirred single-use bioreactor family (BIOSTAT^® ^STR, Sartorius Stedim Biotech, Germany) has design criteria similar to conventional reusable systems. The bioreactors have a cylindrical cultivation chamber, two impellers mounted on a rigid shaft and a submerged sparger. The H/D ratio of 2:1 and the impeller to bag ratio of 0.38 was kept constant for all scales [4]. There is the possibility to select between the impeller configuration 2 × 3-blade segment impeller (downward mixing) and 6-blade disk (bottom) + 3-blade segment (top) impeller. For the process engineering characterization 2 × 3-blade segment impellers were used. The aeration was performed by a combi sparger, which consists a ring sparger part (hole diameter 0.8 mm) and a micro sparger part (hole diameter 0.15 mm).

## Process engineering characterisation

### Design space approach

The field of application of the stirred single-use bioreactor family is the cultivation of mammalian cells. To verify the single-use bioreactors a modern CHO process was considered with a peak cell density of 27 - 28 × 10^6 ^cells/mL. This process defines the key process parameters relevant for the design space definition [3,5], which are a moderate shear rates (tip speeds < 2.0 m/s), a sufficient oxygen transfer rate (k_L_a > 7 h^-1^, supply pure oxygen assumed), a suitable homogeneity (mixing times < 60 s) and a power input per volume (P/V_L_) between 10 and 250 W/m^3 ^(from lab to production scale).

### Power input per volume

**Figure 1:**
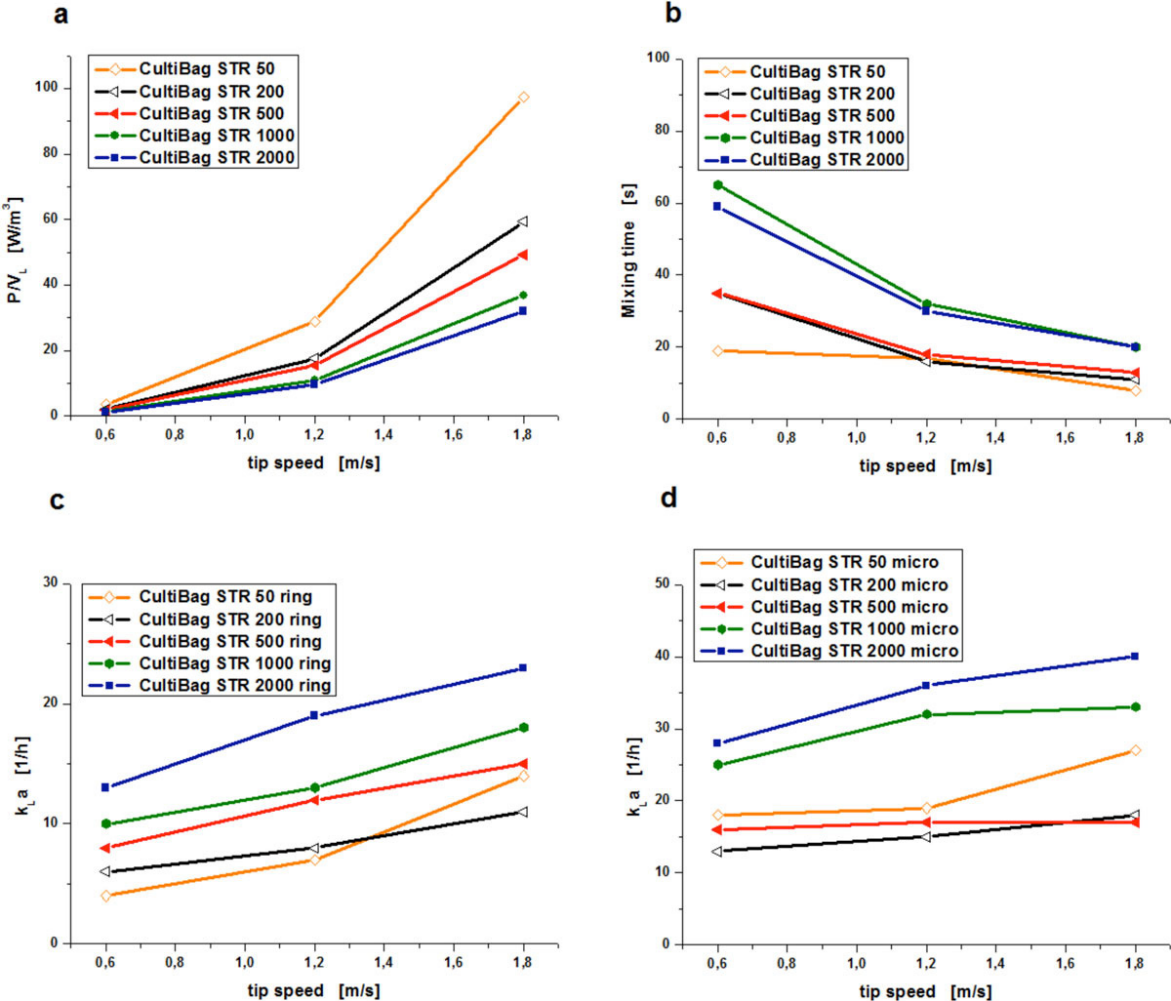
**Process engineering parameters of the CultiBag STR family**. **(a) **Characteristics of the power input per volume, **(b) **mixing time characteristics, **(c) **k_L_a-values for the aeration with the ring sparger part, **(d) **k_L_a-values for the aeration with the micro sparger part.

Energy has to be transferred to a bioreactor to ensure cell suspension, homogenization and gas dispersion [6]. For the quantification the dimensionless Newton number (Ne) was determined by torque measurements [3]. From the results the power input per volume was calculated for tip speeds between 0.6 and 1.8 m/s. Ne for the selected configuration was 1.3. Figure 1a shows the P/V_L _characteristics, which increased for all scales with the tip speed. With increasing size of the CultiBag STR the power input per volume decreases at a defined tip speed.

### Mixing time

Appropriate mixing is important to avoid concentration or temperature gradients inside the cultivation chamber. The mixing time of the stirred single-use bioreactor was determined by the decolourization method [7]. The mixing times as a function of the tip speed are illustrated in Figure 1b. As the tip speed increases, expectedly the mixing times decrease. For all scales mixing times below 30 s are achieved.

### Oxygen transfer capabilities

The oxygen transfer efficiency of a bioreactor can be described by the k_L_a-value, which was determined by the gassing-out method (1xPBS, room temperature) [8]. The aeration was carried out through the holes with 0.8 mm (ring sparger part) (Figure 1c) and in another trial through the holes with 0.15 mm diameter (micro sparger part) (Figure 1d). The volumetric mass transfer coefficients were determined as a function of the tip speed for a constant gas flow rate of 0.1 vvm. With increasing tip speed the k_L_a-value characteristics increased for all scales. For larger scales higher k_L_a-values were achieved presumably due to longer residence times of the gas bubbles. By using aeration through the holes with the smaller diameter the k_L_a-value can be significantly increased.

## Conclusions

The main application of the presented single-use bioreactor family is the cultivation of mammalian and insect cells. These cells have special demands on the cultivation system for their optimal growth. To verify the suitability of the bioreactor family different process engineering parameters were determined. Based on the results the process engineering parameters are in the desired ranges of the defined design space regarding the power input per volume, mixing efficiency and the k_L_a-value. This indicates that the stirred single-use bioreactor family is suitable for cell culture applications. The design criteria of the CultiBag STR family directly relates to those from reusable systems. Therefore, existing challenges for a scale-up or process transfer are removed due to the improved design. Consequently, this technology represents an important step towards further maturity of single-use bioreactors and their acceptance.
